# Oral drug delivery systems for companion animals: an integrative review of medicated treats, chewable tablets, and other pharmaceutical formulations

**DOI:** 10.29374/2527-2179.bjvm004226

**Published:** 2026-06-26

**Authors:** Márcio Gonçalves dos Santos, Joacir Ferreira Andrade, Valker Araujo Feitosa

**Affiliations:** 1 Instituto Federal do Paraná, Colombo, Colombo, PR, Brazil; 2 Universidade Estadual do Centro-Oeste, Guarapuava, PR, Brazil; 3 Faculdade de Ciências Farmacêuticas, Universidade de São Paulo, SP, Brazil

**Keywords:** drug delivery systems, chewable medication, medication adherence, pharmaceutical dosage forms, sistemas de administração de medicamentos, medicamentos mastigáveis, adesão terapêutica, preparação farmacêutica

## Abstract

Administering oral medications to companion animals remains a major challenge in veterinary medicine, frequently leading to poor adherence and reduced therapeutic effectiveness. Medicated treats have emerged as a promising strategy to facilitate drug administration by combining active pharmaceutical ingredients with highly palatable food-based matrices. This integrative review critically synthesized the available scientific literature on oral drug delivery systems for dogs and cats, with emphasis on medicated treats, chewable formulations, therapeutic efficacy, safety, stability, and regulatory challenges. A structured search was conducted in PubMed, SciSpace, Consensus, and Google Scholar, using PRISMA-based procedures. After screening and eligibility assessment, 20 studies were included in the qualitative synthesis, including one additional eligible study incorporated after peer-review recommendation and assessed according to the same predefined criteria. The reviewed studies indicate that medicated treats and chewable oral formulations may improve treatment adherence, reduce administration-related stress, and maintain acceptable pharmacological performance when appropriate formulation parameters are met. Evidence also suggests that formulation-related factors, such as matrix composition and concomitant administration conditions, may influence bioavailability and product performance. Nevertheless, relevant challenges remain regarding dose uniformity, physicochemical stability, microbiological safety, and regulatory classification. Overall, medicated treats represent a promising alternative drug delivery strategy in veterinary therapeutics, particularly for chronic treatments in companion animals. Further studies are needed to optimize formulation parameters, evaluate long-term safety, and establish clearer regulatory frameworks for these hybrid pharmaceutical–nutritional products.

## Resumo

A administração de medicamentos por via oral em animais de companhia ainda representa um desafio relevante na prática veterinária, frequentemente associado à baixa adesão ao tratamento e à redução da eficácia terapêutica. Nesse contexto, os petiscos medicamentosos têm emergido como uma estratégia promissora para facilitar a administração de fármacos, ao combinarem princípios ativos farmacológicos com matrizes alimentares altamente palatáveis. Esta revisão integrativa teve como objetivo analisar a literatura científica sobre sistemas de liberação oral de medicamentos para cães e gatos, com ênfase em petiscos medicamentosos, formulações mastigáveis, eficácia terapêutica, segurança, estabilidade e desafios regulatórios. Foi realizada uma busca estruturada nas bases de dados PubMed, SciSpace, Consensus e Google Scholar, seguindo os procedimentos metodológicos das diretrizes PRISMA. Após as etapas de triagem e avaliação de elegibilidade, 20 estudos foram incluídos na síntese qualitativa. Os resultados indicam que os petiscos medicamentosos podem melhorar a adesão ao tratamento, reduzir o estresse associado à administração de medicamentos e manter um desempenho farmacológico satisfatório quando adequadamente formulados. Entretanto, persistem desafios relacionados à uniformidade de dose, à estabilidade físico-química das formulações e à classificação regulatória desses produtos híbridos. De modo geral, os petiscos medicamentosos constituem uma alternativa promissora para a liberação de fármacos na terapêutica veterinária, especialmente em tratamentos crônicos de animais de companhia. Estudos adicionais são necessários para otimizar parâmetros de formulação, avaliar a segurança em longo prazo e estabelecer marcos regulatórios mais claros para esses sistemas farmacêutico-nutricionais.

## Introduction

Administering oral medications to companion animals is frequently associated with practical and behavioral challenges that may compromise treatment adherence. Dogs and cats often resist conventional dosage forms, such as tablets, capsules, and oral suspensions, which can lead to incomplete dosing, increased stress for both animals and pet owners, and reduced therapeutic success. Improving the acceptability of oral medications is therefore an important objective in veterinary pharmacotherapy (Adenot & Abdelhakim, 2022).

With the growing humanization of companion animals, the global pet market has increasingly invested in value-added products that combine therapeutic functionality, nutrition, and well-being. According to Calancea et al. (2024), treats currently account for 15% of the pet food sector, with annual growth exceeding 10%. This expansion is driven by pet owners’ demand for more natural and palatable products associated with health promotion, which has encouraged the development of medicated and nutraceutical treats (Freiberga et al., 2025).

In this context, drug-incorporated treats have emerged as an innovative strategy that combines voluntary acceptance, masking of unpleasant drug taste, and controlled oral delivery (Taenzler et al., 2017). In addition to facilitating administration, these formulations may promote greater adherence and improve the experience for both animals and pet owners by reducing stress and the risk of incorrect dosing.

The development of chewable veterinary dosage forms requires compliance with strict parameters related to bioavailability, efficacy, safety, and sensory acceptability. According to Kongara and Chambers (2018), the adaptation of active compounds into palatable veterinary formulations must consider species-specific sensory preferences, since dogs and cats differ substantially in taste perception and feeding behavior. Dogs, for example, have taste receptors that are highly sensitive to meat and fatty compounds, whereas cats may respond better to pasty textures and intense aromas (Adenot & Abdelhakim, 2022).

Given these challenges, medicated treats have been increasingly investigated as palatable oral delivery systems that combine active pharmaceutical ingredients with food-based matrices to support voluntary ingestion and improve treatment adherence. A study by Costa et al. (2025) involving 60 dogs reported higher acceptance rates for food-associated dosage forms, such as treats (95%) and pastes (90%), compared with conventional pharmaceutical forms, including capsules (35%) and oral suspensions (60%). These findings suggest that integrating pharmacological therapy with feeding behavior may reduce administration-related stress and improve adherence to treatment (Banach & Ferrero, 2023; Johnson et al., 2020; Ohira et al., 2025).

Food-based matrices may also influence intestinal absorption and product performance, particularly when lipophilic compounds are incorporated into palatable vehicles. In some cases, this approach may enhance absorption and reduce adverse reactions, especially in small dogs (Limsuwan et al., 2024). In the pharmacotechnical field, recent advances include the use of orodispersible films (Koh et al., 2021), lipid nanoemulsions (Limsuwan et al., 2024), and stabilized gelatin bases (Líos et al., 2024), expanding the possibilities for developing chewable and semi-solid controlled-release systems. Formulation attributes such as texture and aroma have also been explored as modulators of palatability, with potential implications for voluntary intake.

From an ecological and nutritional perspective, recent literature indicates a global movement toward sustainability in the production of functional treats. The use of insect proteins, such as *Tenebrio molitor*, fish by-products, plant-based proteins, and prebiotic fibers has been associated with both environmental and nutritional advantages, expanding the potential for innovation in the veterinary industry (Freiberga et al., 2025; Kępińska-Pacelik et al., 2023).

Despite these advances, the incorporation of bioactive compounds into treats still faces methodological and regulatory challenges. Calancea et al. (2024) report a lack of standardized protocols for assessing palatability, stability, and clinical efficacy, which limits comparison across studies and the establishment of quality parameters. Similarly, Líos et al. (2024) emphasize the need for standardized microbiological and physicochemical protocols, particularly for compounded or artisanal formulations, which may present greater variability in moisture, texture, and shelf life.

The scientific literature on medicated treats remains fragmented, encompassing studies from veterinary pharmacology, food technology, pharmaceutical sciences, and animal nutrition. A comprehensive synthesis of current evidence is therefore necessary to clarify the therapeutic potential, formulation challenges, and regulatory implications of these hybrid pharmaceutical–nutritional systems.

**Figure 1:**
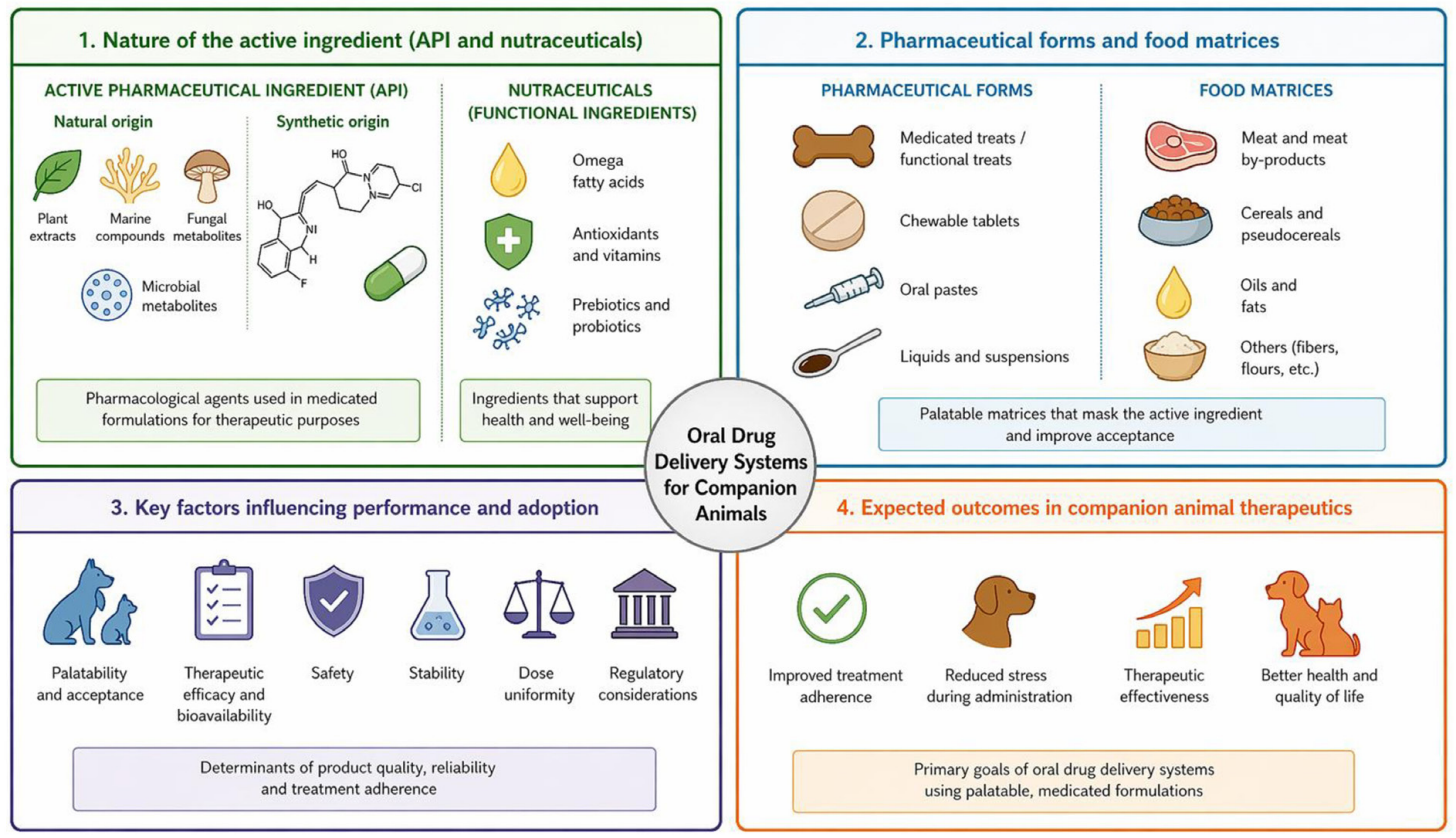
Conceptual framework of oral drug delivery systems for companion animals.

The figure 1 synthesizes the main elements discussed in this review, including the nature of active ingredients (active pharmaceutical ingredients – APIs – of natural or synthetic origin, and nutraceutical compounds), the pharmaceutical dosage forms and palatable food matrices used for oral administration, and the key factors influencing product performance, such as palatability, bioavailability, stability, safety, and dose uniformity. These interconnected components contribute to improved treatment adherence, reduced stress during administration, and enhanced therapeutic effectiveness in veterinary medicine.

Thus, this integrative review aimed to critically synthesize findings from 20 studies published between 2015 and 2025 in order to systematize the available scientific evidence on the efficacy, safety, stability, and acceptance of veterinary drugs and nutraceutical compounds incorporated into dog and cat treats, including medicated treats, chewable tablets, biscuits, pastes, semi-solid treats, and food-based matrices. The review also sought to identify technological trends, scientific gaps, and methodological directions to support the development of innovative and sustainable formulations focused on therapeutic adherence and animal welfare.

## Materials and methods

This study was designed as an integrative review conducted with systematic procedures to synthesize and critically analyze the available scientific evidence on medicated and functional treats used as oral drug delivery systems in veterinary medicine. The integrative review method allows the inclusion of studies with different methodological designs, enabling a comprehensive understanding of emerging therapeutic approaches (Whittemore & Knafl, 2005).

To ensure methodological transparency and reproducibility, the search, screening, and selection procedures were conducted following the recommendations of the Preferred Reporting Items for Systematic Reviews and Meta-Analyses (PRISMA 2020) guidelines (Page et al., 2021).

Unlike traditional systematic reviews, integrative reviews allow the inclusion of studies with different research designs, such as clinical trials, observational studies, and laboratory experiments, integrating quantitative and qualitative findings to produce interpretative inferences and identify scientific gaps (Souza et al., 2010; Whittemore & Knafl, 2005). This type of review is particularly suitable for emerging areas of veterinary pharmacology, where the body of evidence is diverse, fragmented, and still under consolidation, as observed in the field of palatable oral therapeutic systems.

Thus, the present study aimed to systematize, critically analyze, and integrate scientific evidence published between 2015 and 2025, seeking to answer the following guiding question:

“What is the scientific evidence on the efficacy, safety, stability, and acceptance of veterinary drugs incorporated into treats for dogs and cats?”

### Information sources and search strategy

A systematic and comprehensive search was conducted in PubMed, SciSpace, Consensus, and Google Scholar, covering the period from 2015 to 2025, with no language restrictions. Three conceptual axes were explored: (1) animal population, (2) pharmaceutical intervention, and (3) outcomes of interest.

The following descriptors and Boolean operators were used:

(“veterinary drug” OR “veterinary medicine”) AND (“medicated treats” OR “functional snacks” OR “palatable formulations”) AND (“dogs” OR “cats”) AND (“efficacy” OR “safety” OR “stability” OR “acceptance” OR “palatability”).

In addition to the electronic search, a manual search was performed in the reference lists of the included articles to identify relevant publications not retrieved through the primary search strategy.

The electronic searches initially identified 216 records, distributed as follows: PubMed (n = 89), SciSpace (n = 52), Consensus (n = 41), and Google Scholar (n = 34). In addition, two records were identified through manual cross-reference searching.

After completion of the original screening process, one additional potentially relevant study suggested during peer review was assessed using the same eligibility criteria applied in the review. As the study met the predefined inclusion criteria, it was incorporated into the final qualitative synthesis.

### Eligibility criteria

Eligibility criteria were defined using the PICOS framework, originally described by Richardson et al. (1995), and adapted for database search strategies as described by Schardt et al. (2007).

Population (P): Studies involving dogs (*Canis lupus familiaris*) and/or cats (*Felis catus*), with no restrictions regarding breed, age, sex, or health condition.
Intervention (I): Administration of one or more active pharmaceutical ingredients incorporated into a palatable vehicle for oral administration, such as treats, biscuits, chews, chewable tablets, pastes, gels, or any other solid or semi-solid form designed for voluntary consumption.
Comparison (C): Studies comparing the intervention with: (1) administration of the same drug in conventional pharmaceutical forms, such as tablets, capsules, or oral suspensions; (2) a palatable placebo without the drug; or (3) studies without a direct comparator group, provided that they quantitatively assessed at least one outcome of interest, such as acceptance or palatability.
Outcomes (O): Studies reporting at least one of the following primary or secondary outcomes were included:
*Acceptance/Palatability:* voluntary consumption, acceptance rate, palatability scores, and preference tests. Palatability criteria were defined according to recommendations from the Committee for Medicinal Products for Veterinary Use (CVMP) of the European Medicines Agency (EMA), which recommends, for dogs, consumption of ≥80% within the test window. However, no universally standardized method is currently available for designing and comparing palatability trials, which limits meta-analytical approaches.
*Efficacy/Compliance:* ease of administration reported by pet owners, administration success rate, and adherence to treatment.
*Stability*: physicochemical stability, including drug content, content uniformity, and degradation profile, and/or microbiological stability of the product over time.
*Safety:* incidence and description of adverse reactions, such as vomiting, diarrhea, or allergic reactions, associated with administration of the medicated treat.
Study Design (S*):* Randomized and non-randomized clinical trials, observational studies, including cohort and case-control studies, experimental stability studies, and case series were included. Literature reviews, editorials, letters to the editor, and opinion articles were excluded.

### Selection of studies

The search results were imported into Zotero software, which was used to remove duplicate records. After removal of 123 duplicates, 95 unique records remained for screening. Two independent reviewers (Santos and Feitosa) performed the screening in two phases: (i) title and abstract screening, to exclude studies that did not meet the eligibility criteria; and (ii) full-text assessment, to confirm inclusion based on the PICOS criteria. Disagreements were resolved by consensus.

The literature search identified 216 records retrieved from four electronic sources and two additional records obtained through manual cross-reference searching. After removal of 123 duplicates, 95 unique records were screened by title and abstract. Of these, 37 records were excluded because they were outside the scope of the review or did not meet the PICOS criteria. The full texts of 58 articles were then assessed for eligibility, and 39 were excluded after full-text review. Following peer-review recommendation, one additional full-text article was assessed using the same predefined eligibility criteria and was considered eligible for inclusion. Therefore, 20 studies were included in the final qualitative synthesis.

**Figure 2:**
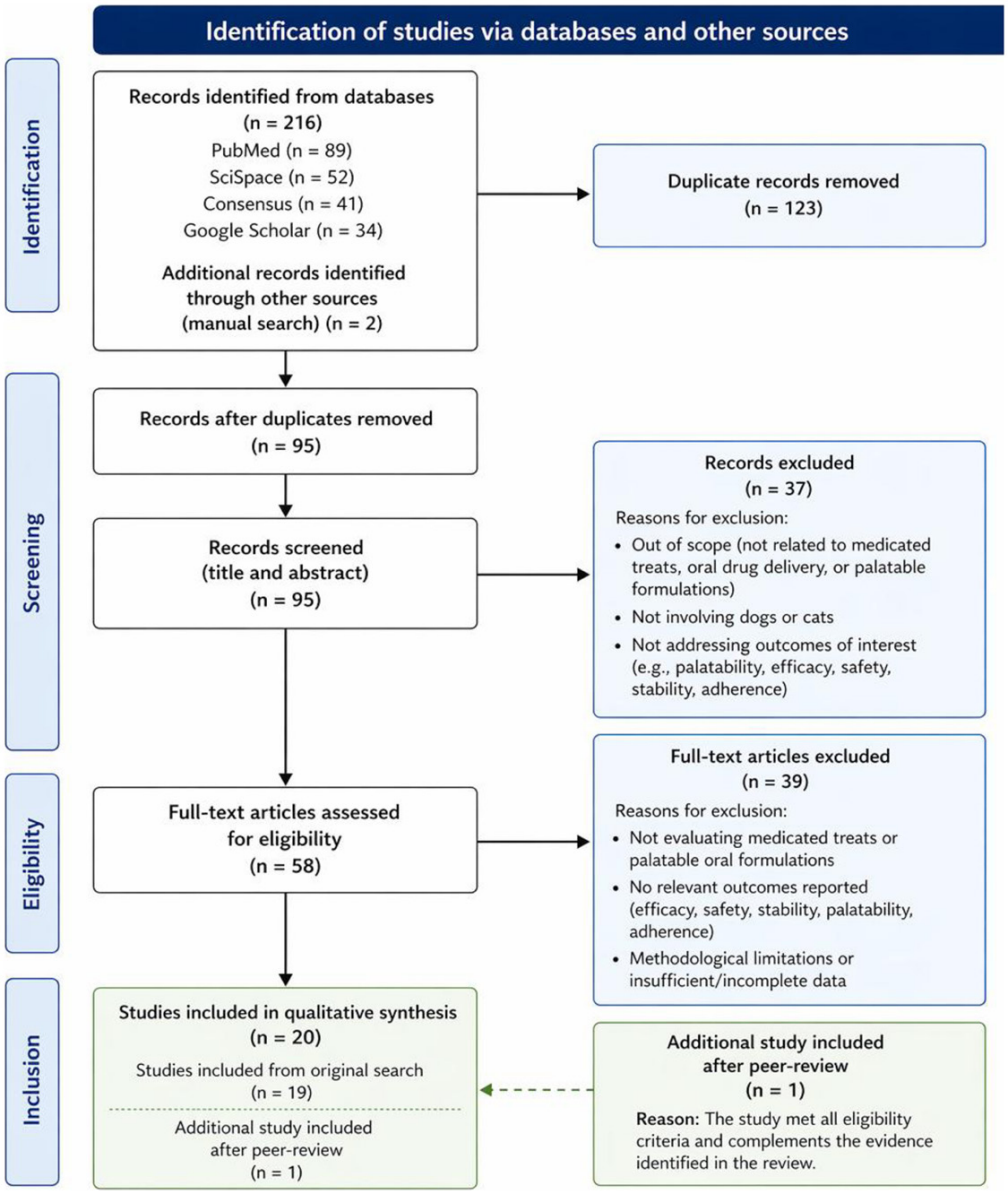
PRISMA 2020 flow diagram of the study selection process.

All counts reported in the PRISMA 2020 flow diagram were cross-checked against the screening spreadsheet and the supplementary PRISMA summary to ensure internal consistency. The study selection process is summarized in the PRISMA 2020 flow diagram presented in Figure 2.

PRISMA 2020 flow diagram of the study selection process used in this integrative review. The diagram summarizes the identification, screening, eligibility assessment, and inclusion of studies investigating medicated and functional treats as oral drug delivery systems for dogs and cats.

### Data extraction

Data extraction was performed independently by two reviewers using a standardized and pre-tested spreadsheet developed in Microsoft Excel/Google Sheets.

The extracted variables included: study identification, including authors, year, and country; study characteristics, including design and objectives; population characteristics, including species, number of animals, age, and sex; intervention characteristics, including active ingredient, dose, and type of treat or palatable vehicle; comparator, including presence and type of control group; and outcomes, including quantitative and qualitative findings related to acceptance, efficacy, stability, and safety.

### Data synthesis

Given the heterogeneity of study designs, formulations, and outcome measures identified in the included studies, a narrative synthesis approach was adopted. The extracted information was organized into thematic categories addressing key aspects of medicated treats, including formulation strategies, palatability and acceptability, therapeutic effectiveness, safety considerations, bioavailability, pharmacokinetics, stability, quality control, and regulatory perspectives.

The synthesis aimed to identify convergent findings, methodological limitations, and emerging research directions related to the development of palatable drug delivery systems for companion animals.

### Assessment of methodological quality and risk of bias

The methodological quality of the included studies was assessed through qualitative appraisal based on study design characteristics, clarity of methodological description, and consistency of reported outcomes. Particular attention was given to sample size, description of experimental procedures, palatability assessment methods, formulation reporting, and transparency of outcome reporting.

Because the included studies presented heterogeneous designs, a narrative assessment of potential sources of bias was conducted instead of applying a quantitative scoring system. Potential sources of bias included small sample sizes, absence of control groups, incomplete reporting of formulation methods, and variability in palatability assessment protocols. This qualitative appraisal supported the identification of methodological limitations that may influence interpretation of the findings.

### Analytical framework

The data were analyzed qualitatively and integratively according to the steps proposed by Whittemore and Knafl (2005). Based on the 20 included studies, the findings were organized into the following analytical axes:

1. acceptance and palatability;
2. efficacy and safety;
3. stability and quality control;
4. bioavailability and pharmacokinetics;
5. innovation and sustainability.

Additionally, a qualitative SWOT analysis, covering strengths, weaknesses, opportunities, and threats, was conducted to synthesize the strategic implications of the findings identified in the reviewed studies.

### Use of artificial intelligence tools

The authors declare that artificial intelligence (AI) tools were used to support specific stages of this study, in accordance with CNPq Ordinance No. 2,664/2026. Consensus and SciSpace were used during the literature search to map references, identify patterns and connections across studies, and explore research gaps. NotebookLM was used for article note-taking, comparative matrices, and comparison of arguments, methods, and alignment with the study objective. ChatGPT was used to assist with the initial manuscript structure, grammar revision, and figure development. All AI-assisted outputs were critically reviewed, revised, and validated by the authors, who assume full responsibility for the originality, accuracy, and integrity of the final manuscript.

## Results

This integrative review with systematic procedures included 20 studies published between 2015 and 2025, comprising clinical trials, observational investigations, and experimental studies related to medicated and functional treats used as oral drug delivery systems for companion animals. The studies were conducted primarily in Europe, North America, and Asia.

Evidence from the selected articles was organized into five analytical axes: (1) acceptance and palatability, (2) efficacy and safety, (3) stability and quality control, (4) bioavailability and pharmacokinetics, and (5) innovation and sustainability. These thematic categories were defined based on the most frequently reported outcomes and methodological characteristics of the included studies.

Overall, the studies evaluated multiple pharmaceutical and nutraceutical compounds administered through palatable vehicles, including chewable tablets, biscuits, pastes, semi-solid treats, and food-based matrices.

### Acceptance and palatability

Acceptance and palatability were the most frequently evaluated outcomes among the included studies. Evidence indicates that the sensory characteristics of the formulation, including flavor, aroma, and texture, may influence voluntary consumption and treatment adherence in dogs and cats.

Dogs generally showed higher acceptance of chewable formulations with meat-based flavor profiles, whereas cats demonstrated greater acceptance of flavored pastes and semi-solid preparations (Adenot & Abdelhakim, 2022; Visser et al., 2022). Differences in taste perception between species were also reported, with dogs possessing approximately 1,700 taste buds compared with approximately 470 in cats, which may influence dietary selectivity (Visser et al., 2022).

Controlled palatability studies demonstrated that complex combinations of flavoring agents, including odor, taste, and mouthfeel, can increase voluntary consumption (Aleo et al., 2018). However, excessively chewy textures were associated with lower complete intake rates, even when strong flavoring agents were used (Aleo et al., 2018).

The study by Costa et al. (2025) found that food-associated dosage forms, such as biscuits and pastes, exhibited notably higher voluntary consumption rates in dogs, 95% and 90%, respectively, compared with conventional forms such as capsules, 35%, and oral suspensions, 60%. These findings support the interpretation that creamy, palatable formulations are more readily accepted by animals and may improve treatment adherence.

Clinical trials evaluating chewable formulations containing oclacitinib and fluralaner reported acceptance rates above 85% in dogs, with reduced stress during drug administration (Taenzler et al., 2017; Visser et al., 2022). In cats, acceptance rates ranged from 60% to 70%, particularly when formulations had a creamy texture and poultry-based flavoring (Adenot & Abdelhakim, 2022).

Additional evidence was provided by Lima et al. (2021), who developed chewable doxycycline tablets for dogs and reported an acceptance rate of 79.2% in palatability testing, with non-inferiority to placebo chewable tablets. These findings reinforce the potential of chewable dosage forms to improve voluntary intake, even for compounds with intrinsically bitter taste profiles.

Studies evaluating formulation parameters also indicated that products with protein content ≥28% and adequate surface moisture showed improved voluntary consumption (Le Guillas et al., 2024). In addition, glycerinated gelatin matrices were reported to produce flexible textures that favored voluntary intake (Líos et al., 2024).

Despite these findings, heterogeneity in palatability assessment methods was observed across studies, including differences in behavioral scoring systems and acceptance criteria (Adenot & Abdelhakim, 2022).

### Efficacy and safety

Several studies evaluated the therapeutic performance and safety of medicated treats containing pharmacologically active compounds. Chewable formulations containing fluralaner demonstrated prolonged efficacy against *Otodectes cynotis* infestations in dogs and cats, with minimal adverse effects reported during treatment (Taenzler et al., 2017). Similarly, chewable robenacoxib formulations showed anti-inflammatory efficacy comparable to conventional tablet formulations while facilitating administration by pet owners (Kongara & Chambers, 2018).

Cannabinoid-based formulations have also been investigated in companion animals. Studies evaluating cannabidiol (CBD) and cannabidiolic acid (CBDA) reported clinical improvements in conditions such as osteoarthritis-related pain, epilepsy, and anxiety, without relevant hepatic or hematological alterations at doses up to 20 mg/kg/day (Di Salvo et al., 2023; Limsuwan et al., 2024).

In the field of nutraceutical formulations, several compounds demonstrated biological activity when incorporated into food matrices. Catechins derived from Japanese green tea showed anti-inflammatory and antioxidant activity following oral administration (Ohira et al., 2025). Similarly, seaweed extracts derived from *Ascophyllum nodosum* were associated with improvements in oral health parameters and antioxidant responses (Di Cerbo et al., 2017; Johnson et al., 2020).

Across the reviewed studies, adverse reactions were generally mild and infrequent, with occasional reports of gastrointestinal disturbances, such as vomiting or diarrhea. However, the small sample sizes and methodological limitations of these studies require caution when generalizing these findings.

### Stability and quality control

Several studies evaluated the physicochemical stability and quality parameters of medicated treats and palatable pharmaceutical formulations.

Regulatory frameworks for veterinary compounding in Brazil establish quality control parameters for veterinary preparations through Good Practices for Compounding Veterinary Products (BPMV), defined by MAPA Normative Instruction No. 11/2005 and later amended by Normative Instruction No. 41/2014 (Brasil, 2005, 2014). These standards define responsibilities related to prescription validation, compounding procedures, labeling requirements, and quality control testing.

Physicochemical stability studies demonstrated that chewable treats prepared with glycerinated gelatin and crushed-treat matrices maintained stable drug content and structural integrity for up to 30 days under refrigerated conditions (Líos et al., 2024). Similar findings were reported for extruded formulations, which preserved moisture content and structural properties under controlled storage conditions (Calancea et al., 2024).

Microbiological evaluations of compounded veterinary products identified variability in sodium levels and microbial load among formulations, suggesting the need for stricter quality control procedures during production (Gross et al., 2023).

Quality control parameters reported in the literature included organoleptic characteristics, pH, active ingredient concentration, and microbiological purity, which are essential for ensuring formulation stability and safety. In addition, Lima et al. (2021) reported acceptable weight variation and content uniformity for chewable doxycycline tablets, indicating that homogeneous drug distribution can be achieved in palatable chewable formulations when pharmacopeial parameters are considered during development.

### Bioavailability and pharmacokinetics

Pharmacokinetic studies demonstrated that the composition of the food matrix can influence drug absorption and systemic bioavailability.

Lipid-rich treat formulations were associated with increased absorption of lipophilic compounds, whereas extruded matrices were reported to slow the initial rate of absorption. Comparative pharmacokinetic studies of CBD formulations in dogs reported relative bioavailability values of 60% to 70% for lipid nanoemulsions and approximately 35% for semi-solid treat formulations (Limsuwan et al., 2024). Similarly, CBD incorporated into lipid matrices showed sustained absorption profiles and adequate safety parameters (Di Salvo et al., 2023).

Alternative oral dosage forms also demonstrated comparable pharmacokinetic performance. For example, studies comparing furosemide administered through orodispersible films and conventional tablets showed shorter absorption time while maintaining similar systemic exposure (Koh et al., 2021). Similarly, Lima et al. (2021) reported that chewable doxycycline tablets administered to Beagle dogs showed no significant difference in AUC0-t compared with a commercial doxycycline tablet, indicating comparable systemic exposure. The same study also demonstrated that co-administration with a vitamin-mineral supplement reduced relative bioavailability, highlighting the importance of matrix-related and co-treatment-related factors in pharmacokinetic performance.

Studies evaluating natural bioactive compounds also reported rapid absorption and biological activity. Green tea catechins administered orally demonstrated systemic absorption within 1 to 2 hours and modulation of inflammatory markers (Ohira et al., 2025). In addition, the incorporation of insect proteins and flaxseed oil into formulations increased the absorption of antioxidants and carotenoids (Kępińska-Pacelik et al., 2023).

### Innovation and sustainability

Several studies reported advances in formulation strategies aimed at improving the sustainability and functional performance of veterinary formulations.

The incorporation of alternative protein sources, such as *Tenebrio molitor*, algae, and plant-derived ingredients, has been explored as a strategy to reduce environmental impact while maintaining the nutritional and functional properties of veterinary treats (Freiberga et al., 2025; Kępińska-Pacelik et al., 2023).

Technological innovations, such as nanoencapsulation, lipid emulsions, and orodispersible films, have been investigated as methods to improve the stability, drug release control, and acceptability of oral veterinary formulations (Le Guillas et al., 2024; Líos et al., 2024).

Emerging concepts, such as digitally traceable formulations and environmentally responsible manufacturing processes, have also been described in the context of “smart treats,” integrating therapeutic functionality with sustainable production strategies (Calancea et al., 2024).

## Discussion

This review underscores the potential of medicated and functional treats as a viable strategy to improve oral drug administration in companion animals. The reviewed studies show that integrating pharmacologically active ingredients into palatable formulations may improve voluntary intake, which is central to treatment adherence. These findings reinforce the importance of palatability as a determinant of successful drug delivery.

### Palatability as a determinant of treatment adherence

One of the most relevant findings identified in the reviewed studies concerns the role of palatability in treatment adherence. Unlike human patients, companion animals cannot understand the therapeutic need for medications, which makes voluntary acceptance a central factor in treatment success. The reviewed evidence demonstrates that oral formulations incorporated into palatable food matrices substantially improve voluntary consumption compared with conventional dosage forms, such as capsules or tablets (Adenot & Abdelhakim, 2022; Aleo et al., 2018; Visser et al., 2022).

Species-specific differences were also observed. Dogs generally demonstrated higher acceptance of chewable treats with meat-based flavor profiles, whereas cats showed greater acceptance of semi-solid formulations, such as pastes or creamy preparations (Adenot & Abdelhakim, 2022; Visser et al., 2022). These differences may be related to species-specific sensory perception, including the number of taste receptors and behavioral feeding patterns (Visser et al., 2022).

The observational study conducted by Costa et al. (2025) supports this interpretation by demonstrating markedly higher voluntary consumption rates for food-associated dosage forms compared with traditional pharmaceutical presentations. These findings highlight the importance of sensory formulation parameters, including flavor, odor, texture, and mouthfeel, in determining the acceptability of veterinary oral formulations.

### Therapeutic performance and clinical implications

Beyond improving voluntary intake, several studies demonstrated that medicated treats can maintain adequate pharmacological performance when properly formulated. Clinical trials involving chewable formulations of fluralaner and oclacitinib showed therapeutic efficacy comparable to that of conventional pharmaceutical forms, while also facilitating drug administration by pet owners (Taenzler et al., 2017; Visser et al., 2022).

This interpretation is further supported by Lima et al. (2021), who demonstrated that a chewable doxycycline tablet for dogs achieved pharmacokinetic exposure comparable to that of a marketed formulation while maintaining favorable palatability. Their findings are particularly relevant because they suggest that improved acceptability does not necessarily require a trade-off with systemic drug exposure, although concomitant supplementation may alter bioavailability.

Similarly, nutraceutical and bioactive compounds incorporated into food matrices demonstrated relevant biological effects. The anti-inflammatory and antioxidant activity observed with green tea catechins and seaweed-derived compounds suggests that food-based delivery systems may support pharmacological treatment and provide functional nutritional benefits (Di Cerbo et al., 2017; Johnson et al., 2020; Ohira et al., 2025). However, the limited number of studies and variations in study design suggest that further research is needed to confirm the clinical benefits of these nutraceuticals in companion animals.

The therapeutic responses observed across different classes of bioactive compounds suggest that food-based delivery systems may extend beyond drug administration and support multifunctional clinical outcomes. In this context, incorporating pharmacological and nutraceutical agents into palatable matrices may enable combined therapeutic and supportive effects, particularly in chronic conditions (Di Salvo et al., 2023; Limsuwan et al., 2024). However, the heterogeneity of study designs and outcome measures limits the generalizability of these findings and underscores the need for more standardized clinical evaluations.

Nevertheless, the long-term safety and efficacy of cannabinoid-based formulations in veterinary medicine remain unclear, requiring additional studies with larger sample sizes and more consistent methodologies before definitive conclusions can be drawn. These findings suggest the therapeutic potential of integrating pharmacological compounds into palatable delivery systems, particularly for chronic conditions requiring prolonged treatment, but further investigation is needed to fully assess their benefits and risks.

### Technological challenges in formulation

Although the reviewed studies demonstrate the potential of medicated treats, challenges related to dose consistency and formulation stability continue to pose significant obstacles. Variations in active ingredient distribution, particularly in food-based matrices, can compromise dose uniformity, which remains a major challenge in formulation development. In addition, the physicochemical stability of these treats is crucial for ensuring their effectiveness over time, especially in long-term treatments.

Studies evaluating compounded treats have shown that temperature, humidity, and matrix composition may influence degradation rates and overall formulation stability (Calancea et al., 2024; Líos et al., 2024).

Microbiological quality control also represents an important consideration. Investigations evaluating compounded veterinary preparations have identified variability in sodium levels and microbial contamination across products, indicating the need for strict manufacturing and storage protocols (Gross et al., 2023). These factors highlight the importance of adopting standardized formulation and quality control procedures to ensure product safety and therapeutic reliability.

### Pharmacokinetic considerations

Another important aspect discussed in the literature concerns the influence of the food matrix on drug bioavailability. Pharmacokinetic studies suggest that the lipid content and structural characteristics of the treat can significantly influence absorption and systemic exposure of the active compound (Di Salvo et al., 2023; Kongara & Chambers, 2018; Limsuwan et al., 2024).

For example, lipid nanoemulsions demonstrated higher bioavailability of cannabidiol compared with semi-solid treat formulations (Limsuwan et al., 2024). Similarly, lipid-rich matrices were associated with increased absorption of lipophilic compounds, whereas extruded formulations tended to delay initial absorption (Di Salvo et al., 2023).

Alternative oral delivery systems, such as orodispersible films, have also demonstrated favorable pharmacokinetic profiles compared with conventional tablets, suggesting that technological innovations in dosage form design may further improve drug administration in veterinary medicine (Koh et al., 2021).

The study by Lima et al. (2021) adds an additional layer to this discussion by showing that, although chewable doxycycline tablets achieved pharmacokinetic exposure comparable to that of a commercial formulation, co-administration with a vitamin-mineral supplement reduced relative bioavailability. This finding suggests that the performance of palatable oral formulations depends both on the formulation matrix itself and on concurrent dietary or supplemental factors that may interfere with absorption.

### Regulatory considerations

Regulatory ambiguity remains one of the main challenges for medicated treats. Because these products occupy an intermediate position between pharmaceutical dosage forms and functional foods, their classification may vary across regulatory systems. In the United States and Europe, the terms “medicated treats” and “functional medicated treats” are not yet consistently recognized as standardized regulatory categories. In Brazil, compounded veterinary preparations are regulated under Good Practices for Compounding Veterinary Products, established by MAPA Normative Instruction No. 11/2005 and amended by Normative Instruction No. 41/2014. However, the hybrid pharmaceutical-nutritional nature of these products may require more specific guidance on dose uniformity, labeling, stability, microbiological safety, and quality control.

**Figure 3:**
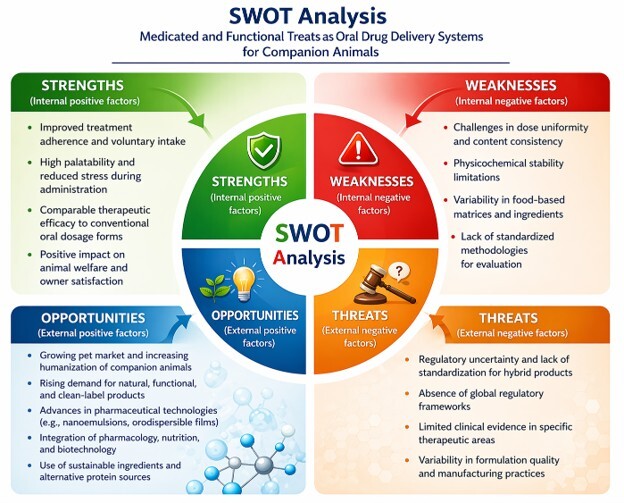
SWOT analysis summarizing the main strengths, weaknesses, opportunities, and threats associated with medicated and functional treats as oral drug delivery systems for companion animals.

Taken together, the evidence identified in the reviewed studies highlights both the potential and the current limitations of medicated treats as veterinary drug delivery systems. To synthesize these aspects, a strategic SWOT analysis was developed based on the findings of this review, summarizing the main strengths, weaknesses, opportunities, and threats associated with the development and application of medicated treats in veterinary medicine (Figure 3).

### Future perspectives for veterinary pharmaceutical innovation

The analyzed studies also indicate growing interest in innovative and sustainable approaches to veterinary drug delivery. The incorporation of alternative protein sources, such as insect-based ingredients and plant-derived bioactive compounds, has been proposed as a strategy to reduce environmental impact while maintaining the functional performance of veterinary formulations (Freiberga et al., 2025; Kępińska-Pacelik et al., 2023).

Technological advances, including nanoencapsulation, lipid emulsions, and novel polymer matrices, may further improve the stability, bioavailability, and palatability of oral veterinary medications (Le Guillas et al., 2024; Líos et al., 2024). In addition, emerging concepts such as digitally traceable formulations and environmentally responsible production processes suggest new directions for the development of hybrid pharmaceutical-nutritional products (Calancea et al., 2024).

Overall, the integration of pharmaceutical technology, veterinary therapeutics, and food science appears to be a promising pathway for improving treatment adherence and therapeutic outcomes in companion animals. However, the heterogeneity of study designs and palatability assessment methods among the included studies may limit direct comparison of results.

An important limitation identified in this review is the predominance of studies conducted in dogs, with comparatively limited evidence available for cats and other companion animals. This imbalance may be partially explained by differences in ease of administration, behavioral compliance, and the greater availability of clinical trials involving dogs. However, species-specific differences in taste perception, feeding behavior, and metabolism may influence both palatability and pharmacokinetic responses, limiting the direct extrapolation of findings across species.

Therefore, caution is required when generalizing these results to cats and other companion animals. Future studies should prioritize the inclusion of diverse species to ensure broader applicability and support the development of more tailored veterinary drug delivery systems.

## Conclusion

The findings indicate that medicated treats represent a feasible alternative for veterinary drug delivery, particularly in clinical contexts requiring prolonged administration. However, their applicability depends on overcoming formulation, standardization, and regulatory challenges that remain insufficiently addressed in the current literature.

The studies analyzed indicate that properly formulated medicated treats may maintain satisfactory pharmacological performance while improving palatability and acceptability. Nevertheless, important challenges remain regarding dose uniformity, physicochemical stability, and quality control, particularly in heterogeneous food-based matrices. In addition, the hybrid nature of these products, positioned between pharmaceutical formulations and functional foods, raises regulatory considerations that require clearer classification and standardization within veterinary regulatory frameworks.

Overall, the integration of veterinary pharmacology, pharmaceutical technology, and food science offers a promising pathway for the development of innovative oral delivery systems for companion animals. Such approaches may be particularly relevant for chronic treatments, in which long-term adherence is essential for therapeutic success.

Future research should focus on optimizing formulation parameters, improving stability and dose standardization, evaluating long-term safety, and establishing clearer regulatory guidelines for these hybrid pharmaceutical-nutritional products. Advances in these areas may contribute to the development of safer, more effective, and more acceptable therapeutic strategies in veterinary medicine.

## References

[B001] Adenot F., Abdelhakim A. (2022). Palatability assessment of oral dosage forms for companion animals: A systematic review. Journal of Drug Delivery Science and Technology.

[B002] Aleo M., Ross S., Becskei C., Coscarelli E., King V., Darling M., Lorenz J. (2018). Palatability Testing of Oral Chewables in Veterinary Medicine for Dogs. Open Journal of Veterinary Medicine.

[B003] Banach D., Ferrero A. (2023). Cannabis and pathologies in dogs and cats: A review. Journal of Cannabis Research.

[B004] Brasil (2005). Regulamento técnico para registro e fiscalização de estabelecimentos que manipulam produtos de uso veterinário (Instrução normativa nº 11, de 8 de junho de 2005)..

[B005] Brasil (2014). Instrução normativa nº 41, de 4 de dezembro de 2014..

[B006] Calancea B.-A., Daina S., Macri A. (2024). The science of snacks: A review of dog treats. Frontiers in Animal Science.

[B007] Costa M. B. F., Chamelete M. O., Martinez M. S. D. S. S., Andrade T. U. (2025). Palatability test of different pharmaceutical forms for administration of continuous-use medications in dogs: Evaluation by owners. Observatório de la Economía Latinoamericana.

[B008] Di Cerbo A., Morales-Medina J. C., Palmieri B., Pezzuto F., Cocco R., Flores G., Iannitti T. (2017). Functional foods in pet nutrition: Focus on dogs and cats. Research in Veterinary Science.

[B009] Di Salvo A., Conti M. B., Della Rocca G. (2023). Pharmacokinetics, efficacy, and safety of cannabidiol in dogs. Frontiers in Veterinary Science.

[B010] Freiberga A., Ilgaza A., Strausa E., Ciprovica I., Zagorska J. (2025). Snacks and ice cream as complementary dog feed: Perspectives, trends, ingredients. Frontiers in Animal Science.

[B011] Gross J., Weber J., Gnoatto F. L. C., Champion T. (2023). Determinação do teor de sódio de petiscos caninos e comprimidos palatáveis manipulados para cães. Estudos Avançados sobre Saúde e Natureza.

[B012] Johnson K. A., Lee A. H., Swanson K. S. (2020). Nutrition and nutraceuticals in the changing management of osteoarthritis for dogs and cats. Journal of the American Veterinary Medical Association.

[B013] Kępińska-Pacelik J., Biel W., Mizielińska M., Iwański R. (2023). Chemical composition and palatability of nutraceutical dog snacks. Applied Sciences.

[B014] Koh S.-K., Jeong J.-W., Choi S.-I., Kim R. M., Koo T.-S., Cho K. H., Seo K.-W. (2021). Pharmacokinetics and diuretic effect of furosemide after single intravenous, oral tablet, and newly developed oral disintegrating film administration in healthy beagle dogs. BMC Veterinary Research.

[B015] Kongara K., Chambers P. (2018). Robenacoxib in the treatment of pain in cats and dogs: Safety, efficacy and place in therapy. Veterinary Medicine.

[B016] Le Guillas C., Vanacker P., Salles C., Labouré H. (2024). Insights to study, understand and manage extruded dry pet food palatability. Animals.

[B017] Lima I. P., Magalhães V. S., Oliveira R. M., Ferreira T. P., Santos G. C. M., Alves M. C. C., Pereira G. A., Silva F. C. S., Rodrigues L. F., Borges D. A., Oliveira P. C., Scott F. B., Cid Y. P. (2021). Development and pharmacokinetic evaluation of chewable doxycycline tablets in Beagle dogs: Comparison with a commercial formulation and evaluation of co-administration with vitamin supplement on the bioavailability. Brazilian Journal of Veterinary Medicine.

[B018] Limsuwan S., Phonsatta N., Panya A., Asasutjarit R., Tansakul N. (2024). Pharmacokinetics behavior of four cannabidiol preparations following single oral administration in dogs. Frontiers in Veterinary Science.

[B019] Líos D. M. C. K., Jiménez A. C., Sandi-Brenes K., Zúñiga Bermúdez S., Pacheco-Artavia M. E., Zavaleta Monestel E. (2024). Desarrollo de tabletas masticables veterinarias. Revista Crónicas Científicas.

[B020] Ohira C., Kaneki M., Shirao D., Kurauchi N., Fukuyama T. (2025). Oral treatment with catechin isolated from Japanese green tea significantly inhibits the growth of *Porphyromonas gulae* and ameliorates gingivitis and halitosis in cats and dogs. International Immunopharmacology.

[B021] Page M. J., McKenzie J. E., Bossuyt P. M., Boutron I., Hoffmann T. C., Mulrow C. D., Shamseer L., Tetzlaff J. M., Akl E. A., Brennan S. E., Chou R., Glanville J., Grimshaw J. M., Hróbjartsson A., Lalu M. M., Li T., Loder E. W., Mayo-Wilson E., McDonald S., McGuinness L. A., Stewart L. A., Thomas J., Tricco A. C., Welch V. A., Whiting P., Moher D. (2021). The PRISMA 2020 statement: An updated guideline for reporting systematic reviews. BMJ).

[B022] Richardson W. S., Wilson M. C., Nishikawa J., Hayward R. S. (1995). The well-built clinical question: A key to evidence-based decisions. ACP Journal Club.

[B023] Schardt C., Adams M. B., Owens T., Keitz S., Fontelo P. (2007). Utilization of the PICO framework to improve searching PubMed for clinical questions. BMC Medical Informatics and Decision Making.

[B024] Souza M. T., Silva M. D., Carvalho R. (2010). Revisão integrativa: O que é e como fazer. Einstein.

[B025] Taenzler J., de Vos C., Roepke R. K. A., Frénais R., Heckeroth A. R. (2017). Efficacy of fluralaner against *Otodectes cynotis* infestations in dogs and cats. Parasites & Vectors.

[B026] Visser M., Walsh K., King V., Sture G., Caneva L. (2022). Acceptance of oclacitinib maleate chewable tablets in client-owned dogs with allergic and atopic dermatitis. BMC Veterinary Research.

[B027] Whittemore R., Knafl K. (2005). The integrative review: Updated methodology. Journal of Advanced Nursing.

